# Enhanced recovery after surgery (ERAS) pathway for primary hip and knee arthroplasty: study protocol for a randomized controlled trial

**DOI:** 10.1186/s13063-019-3706-8

**Published:** 2019-10-22

**Authors:** Jingyi Li, Haibei Zhu, Ren Liao

**Affiliations:** 1 Department of Dermatovenereology, West China Hospital of Sichuan University, Chengdu, 610041 China; 2 Division of Anaesthesiology & Perioperative Medicine, Singapore General Hospital, Outram Road, Singapore, 169608 Singapore; 3 Department of Anesthesiology, West China Hospital of Sichuan University, 37 Guoxue Lane, Chengdu, 610041 China

**Keywords:** Randomized controlled trial, Enhanced recovery after surgery, Total hip arthroplasty, Total knee arthroplasty

## Abstract

**Background:**

With the substantially growing trend of the aging populations in China and the rest of the world, the number of total hip and total knee arthroplasty (THA and TKA) cases are increasing dramatically. It is important to develop practical strategies to improve the quality of healthcare and better outcome for patients undergoing THA and TKA. Enhanced recovery after surgery (ERAS) pathways have been reported to promote earlier recovery and be beneficial for patients. We propose the hypothesis that the ERAS pathway could reduce the length of stay (LOS) in hospital for patients undergoing primary THA or TKA.

**Methods/Design:**

This trial is a prospective, open-labelled, multi-centered, randomized controlled trial that will test the superiority of the ERAS pathway in term of LOS in hospital for the patients undergoing primary THA or TKA compared to current non-ERAS clinical practice. A total of 640 patients undergoing primary THA or TKA will be randomly allocated to either ERAS pathway (ERAS group) or conventional care according to individual participating center (non-ERAS group). The primary outcome is the total LOS in hospital; the secondary outcomes include postoperative LOS, all-cause mortality by 30 days after operation, in-hospital complications, early mobilization, postoperative pain control, total in-hospital cost, and readmission rate by 30 days after discharge from the hospital.

**Discussion:**

This trial is designed to evaluate the superiority of the ERAS pathway to conventional non-ERAS clinical practice in reducing the LOS. The results may provide new insight into the clinical applications of the ERAS pathway for THA and TKA.

**Trial registration:**

National Institutes of Health Clinical Trials Registry, NCT03517098. Registered on 4 May 2018.

## Background

Total joint arthroplasty is the definitive treatment for end-stage osteoarthritis of the hip and knee. The number of the cases increased significantly due to fast-growing aging populations around the world. It was reported that 0.33 million total hip arthroplasty (THA) and 0.7 million total knee arthroplasty (TKA) were performed in the United States annually, and the demand for the procedures were estimated at 0.57 million and 3.48 million per year in 2030, respectively [1]. It is important to establish practical strategies to improve the quality of healthcare and achieve earlier recovery and better outcome for the patients undergoing THA and TKA, while, facilitating a reduction in the heavy healthcare-related economic burden associated with the increasing number of the procedures.

Enhanced recovery after surgery (ERAS) is proposed as a series of evidence-based perioperative optimizations with multidisciplinary approach to reduce surgical stress and accelerate postoperative recovery [2]. Following the general guidelines, different ERAS pathways have been reported to reduce the morbidities, save cost, promote faster recovery, and achieve the clinical and economic gain in colorectal [3], thoracic [4], and orthopedic surgeries [5]. Regional anesthesia is recommended for ERAS because it provides reliable analgesia and little disturbance on hemodynamics in the published literature [2–4, 6]. However, for patients undergoing THA and TKA, spinal anesthesia is frequently associated with prolonged indwelling urinary catheter [7] and, furthermore, the peripheral nerve block may weaken the lower limb muscle strength, leading to delayed mobilization [8].

With the application of short-acting opioid analgesic remifentanil, and anesthetic agents including propofol, sevoflurane, or desflurane, rapid emergence from general anesthesia could be achieved without weakened muscle strength [9]. Based on the pharmacological characteristics of these agents, we propose the hypothesis that general anesthesia-based ERAS with the use of short-acting agents and without the nerve block or intrathecal analgesia could provide reduced length of stay (LOS) in hospital because of the avoidance of weakened muscle strength and possibly early mobilization.

In this trial, we develop an ERAS pathway to compare the conventional care group for patients undergoing primary THA and TKA. The aim of this trial is to scrutinize our hypothesis that ERAS could provide reduced LOS when compare with the current clinical practice.

## Methods/Design

### Trial design

**Fig. 1:**
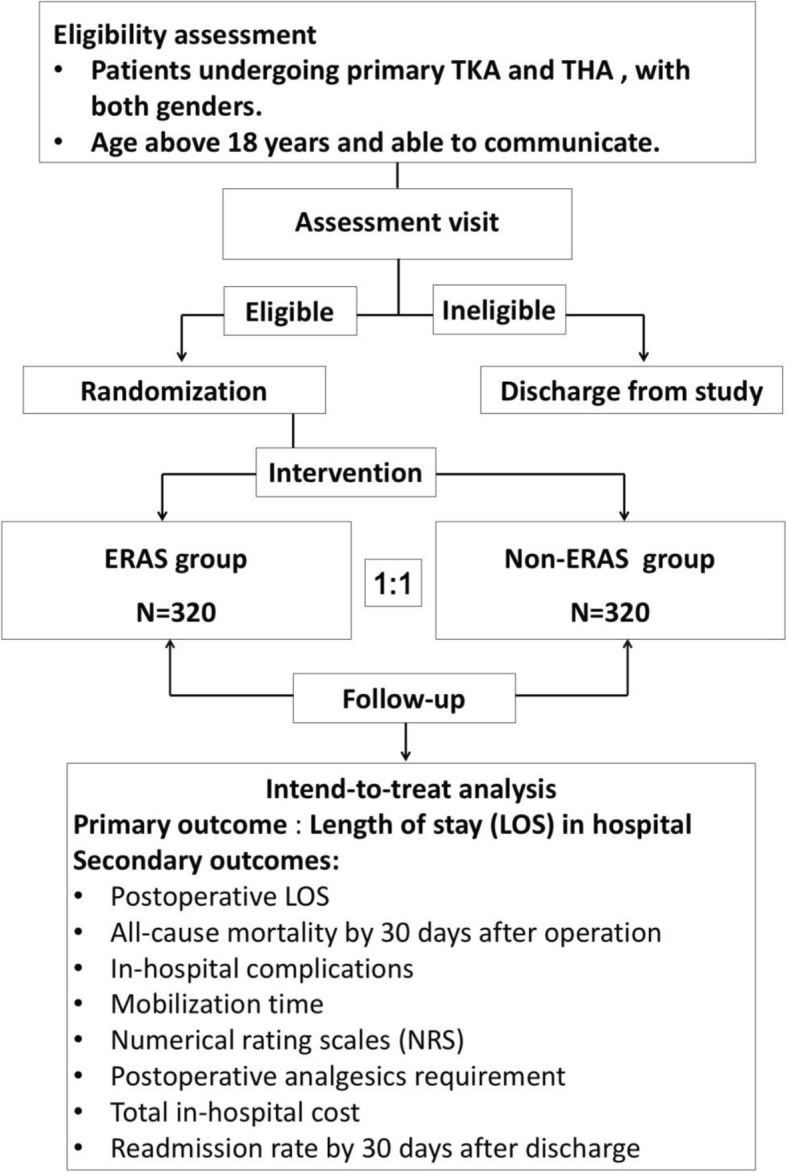
Flow chart for participant eligibility, interventions, assessments, and follow-up

**Fig. 2:**
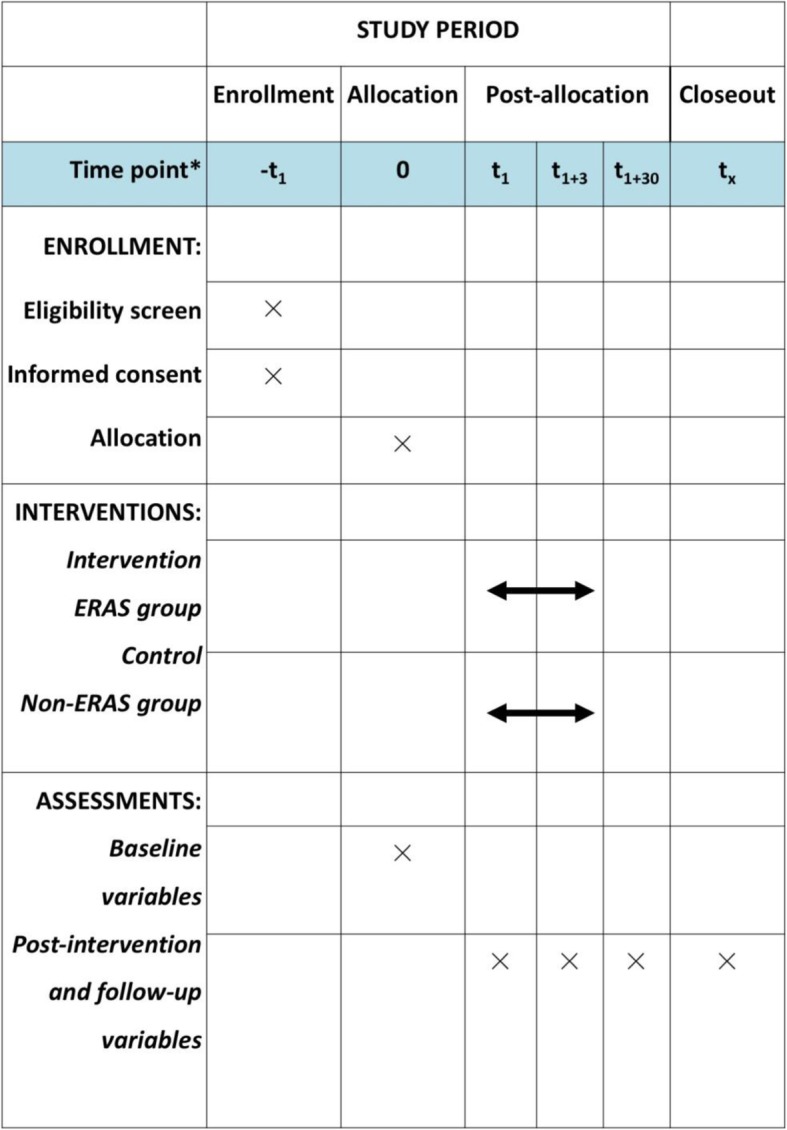
Standard Protocol Items: Recommendations for Interventional Trials (SPIRIT) figure (numbers beside t represent days)

The ERAS trial (http://www.clinicaltrials.gov, registration number: NCT03517098) is a prospective, randomized controlled trial that tests the superiority of the ERAS pathway to current clinical practice in term of reduction of LOS. It will be conducted under the regulations of the Declaration of Helsinki. Following the CONSORT statement (http://www.consort-statement.org/), a brief flow diagram of the ERAS trial is summarized in Fig. 1; a checklist of Standard Protocol Items: Recommendations for Interventional Trials (SPIRIT) is provided in Fig. 2. SPIRIT 2013 Checklist: Recommended items to address in a clinical trial protocol and related documents is provided in Additional file 1.

This trial is supported by a grant from the Key research and development (R&D) Program of Science & Technology Department of Sichuan Province (no. 2019YFS0224) and the National Natural Science Foundation of China (no. 81502722). There is no conflict of interest in the whole process of study execution. A training manual will be produced to educate and train the study team, including orthopedic surgeons, anesthesiologists, nurses, and clinical research coordinators before starting the trial.

### Sample size calculation

Our primary hypothesis is that the application of the ERAS pathway would reduce the LOS in hospital when compared to the current non-ERAS practice in total joint arthroplasty (TJA). According to retrospective analysis of LOS of in hospital of TJA in 2014 (when the ERAS pathway was just proposed) and 2016 (when the ERAS pathway was applied in about 50% of patients) in the Department of Orthopedics, West China Hospital of Sichuan University, the mean LOS of THA was 10 ± 1.5 days in 2014 and 8.6 ± 1.1 days in 2016; the mean LOS of TKA was 12.1 ± 2.1 days in 2014 and 8.9 ± 1.5 days in 2017. For THA, assuming the difference between the two groups was a 5% significance level and a power of 0.90, 17 patients in each group are required; for TKA, assuming the difference between two groups was a 5% significance level and a power of 0.90, only seven patients in each group are required for a comparison within the group. Considering an estimated 20% dropout rate, there should be 42 patients in each group for THA and 20 patients in each group for TKA; a total of 62 patients are required for this study [10]. For a better application of the ERAS pathway for orthopedic surgeons and anesthesiologists, we increased the sample size to 160 individuals in each group for THA and TKA, and a total of 640 individuals will be included. We plan to enroll 320 patients undergoing TKA to participate in this trial in the first stage and enroll 320 patients undergoing THA after completion. A primary comparison will be performed separately for TKA and THA.

### Recruitment

A total of 640 patients undergoing elective arthroplasty, 320 in THA and 320 in TKA, will be enrolled at eight hospitals in China. Individuals will be enrolled and operated in the same hospital even under potentially different conventional clinical practice.

### Randomization and blinding

We will use the Central Randomization System (CRS) to screen and randomize the patients. We will include eight hospitals as participating centers and the centers will be stratified first to guarantee balance between arms within each hospital. According to the sequence of time that the participants are enrolled, after entering the screen number and the individual’s information, the computer-based randomized number prepared on the Internet and allocated group could be retrieved on the website of the CRS. While the individuals, staff responsible for follow-up, and the statisticians will be blinded to the treatment assignment, the investigators, research assistants, and the responsible physicians will not be blinded.

### Study organization and quality control

The principal investigator, study coordinators, and the Office of Scientific Research at West China Hospital are jointly responsible for all aspects of the study protocol and relevant amendments. The implementation of the study, data completeness, and accuracy will be supervised by the Department of Anesthesiology, West China Hospital. The data safety and monitoring board (DSMB), including an orthopedic surgeon, an anesthesiologist, a statistician, a physician, and a member from the Office of Scientific Research who are not unrelated to this trial, will be involved for the entire duration of the trial to review all investigational data for accuracy and completeness periodically to ensure protocol compliance, and to make the decision to stop the trial if there are abnormal frequencies of safety issues, or if the safety of a participant was not guaranteed because of serious complications. Dr. Ren Liao, associated professor of Department of Anesthesiology, West China Hospital, will be the personnel to alert serious complications to the DSMB and be responsible for site monitoring. Data collection and follow-up will be performed by clinical research coordinators. There will be no preliminary analysis performed before the completion of the study.

### Enrollment criteria

#### Inclusion criteria

- Patients undergoing primary TKA and THA, of both genders
- Age > 18 years
- Able to communicate, particularly the ability to express and totally understand an informed consent

#### Exclusion criteria

- Refusal to sign consent
- Pregnancy or lactating female patients
- History or family history of malignant hyperthermia
- Known allergy to propofol, desflurane, or any other anesthetic agent
- History of substance abuse
- History of postoperative delirium
- Impairment of cognitive function or communication
- Psychopathy
- Active participation in another trial where the primary endpoint follow-up is ongoing
- Unwillingness or inability to comply with protocol procedures

### Interventions

#### The ERAS group

Patients will be treated according to the ERAS pathway, which is elaborated below.

ERAS pathway for orthopedic surgeons:

1. Preoperative fasting time: 8 h for consumption of fats and protein food, 6 h for starchy food or non-human milk, and 2 h for clear liquids before operation.
2. Intravenous 20 mg/kg of tranexamic acid will be given 15 min before incision.
3. No indwelling urine catheters.
4. No tourniquet used for TKA.
5. No drainage tube after surgery.
6. Give low molecular heparin subcutaneously 6 h after the operation.

ERAS pathway for anesthesiologists:

1. Before anesthesia induction, ECG, non-invasive blood pressure, pulse oximetry, and capnogram will be continuously monitored for every patient.
2. Intravenous 10 mg of dexamethasone immediately before anesthesia induction.
3. Anesthesia will be induced with 0–0.2 μg/kg of sufentanil or 0–2 μg/kg of fentanyl, 0.3 mg/kg (for THA) or 0.15 mg/kg (for TKA) of cis-atracurium, and 1 mg/kg of propofol. Endotracheal intubation of insertion or laryngeal mask will be performed when the BIS value decreases to 50.
4. Anesthesia will be maintained by titrating continuous remifentanil infusion at a range of 0.15–0.3 μg/kg.min with continuous infusion of propofol of 2–4 mg/kg/h, or keeping end- tidal desflurane (Et-Des) level at 5–7% or sevoflurane (Et-Sev) level at 1.5–2.5%. The BIS value will be kept in the range of 40–60 during the procedure.
5. Incision site will be infiltrated with 40–50 mL of 0.2% ropivacaine at the end of the operation for postoperative analgesia; no patient-controlled intravenous analgesia devices will be applied postoperatively.

#### The non-ERAS (Control) group

Patients undergoing THA or TKA will receive conventional care according to the individual participating center. There is no standard protocol for preoperative management, including fasting guidelines, no restriction of choices of anesthetic techniques and intraoperative medications, as well as postoperative analgesia, and indwelling urinary catheter.

For all the individuals enrolled in the study, no matter which group they are allocated, the decisions regarding whether to place the urinary catheter or drainage tube, or to use tourniquet, or to perform any other medical treatment should be made by the individual responsible physician, based on his clinical judgment. For example, if a patient is allocated to the ERAS group, but he develops urinary retention perioperatively as a result, the urinary catheter could be retained. In such case, we will make a record, and the patient will still be followed up and his data will be collected and analyzed in the ERAS group as per other individuals in the same group.

### Outcome measures

#### Primary outcome

The primary outcome is LOS in hospital, which is defined as the time frame from the day of hospital admission to discharge from the hospital (unit: days).

#### Secondary outcomes

i. Postoperative LOS, which is defined as the time frame from the day of operation to discharge from the hospital (unit: days).
ii. All-cause mortality by 30 days after operation.
iii. In-hospital complications, which are divided into five grades:
  - Grade I: recovery after temporary treatment, e.g. postoperative nausea and vomiting (PONV), postoperative anxiety, insomnia.
  - Grade II: prolonged hospitalization, e.g. pulmonary infection requiring antibiotics or other treatment, surgical wound infection requiring wound debridement.
  - Grade III: life-threatening complications requiring intense treatment during hospitalization and resulting in good functional recovery, e.g. dialysis therapy for acute renal insufficiency, mechanical ventilatory support for respiratory failure, or postoperative bleeding requiring re-operation.
  - Grade IV: life-threatening complications resulting in significantly decreased quality of life, e.g. myocardial infarction, stroke that left paralytic limbs.
  - Grade V: all-cause mortality by 30 days after operation, which is defined as a secondary outcome.
iv. Mobilization time, which is defined as the time frame from the end of operation to the ability to walk without external assistance (unit: hours).
v. Numerical rating scales (NRS) scores at rest during three days after operation.
vi. NRS scores during mobilization or physical therapy during three days after operation.
vii. Postoperative sufentanil or other analgesics requirement during three days after operation.
viii. Total in-hospital cost.
ix. Readmission rate by 30 days after discharge from the hospital.

### Statistical analysis

We plan to execute this trial in two stages, that is, we test the hypothesis in TKA first and then in THA, and the primary comparison will be performed separately for TKA and THA. An intent-to-treat analysis will be performed to analyze all primary and secondary outcomes by using SPSS18.0 software (Statistic Package for Social Science, SPSS, Inc., Chicago, IL, USA). The baseline continuous variables will be summarized using mean ± standard deviation or median (interquartile range) for continuous variables as appropriate. Categorical variables will be summarized using frequency (percentage).

Primary outcome: normality will be assessed for the primary outcome LOS. Appropriate transformation will be made if there is an assumption violation. We will use independent two-sample t-test to compare the LOS between the two groups. A *p* value < 5% will be considered as significant. For the primary outcome, we will also perform a sensitivity analysis using an analysis of covariance model (ANCOVA) with LOS as the response variable, the treatment group as the main factor adjusting for potentially imbalanced baseline characteristics and center effect.

For the primary outcome, we will also perform subgroup analysis based on subgroups defined by each of the age groups (< 65 years, ≥ 65 years), gender (male, female), and American Society of Anesthesiologists (ASA) class (I–II, III, or higher). Analyses will be performed for each subgroup in a similar way to the primary analysis.

For the secondary outcomes, the continuous variables (postoperative LOS, mobilization time, NRS, etc.) will be assessed for normality. Two-sample t-test or Wilcoxon rank sum test will be used for comparison depending on the normality test result. For categorical outcomes, odds ratio will be calculated and tested using Chi-square test. A *p* value < 0.05 will be considered statistically significant. We will use Kaplan–Meier plots to describe the mortality and compare the mortality at 30 days using a log rank test for fixed time points.

## Discussion

The concept of ERAS, or fast-track surgery, was introduced in 1997 by Dr. Henrik Kehlet [2], a gastrointestinal surgeon who won 2014 Excellence in Research Award by the ASA for his outstanding contributions to anesthesiology by the creation of ERAS. A number of studies, including clinical studies [11–13], reviews [6, 14, 15], and meta-analyses [4, 16, 17], demonstrated that compared with conventional treatment, implementation of the ERAS protocol could be associated with reduced LOS in hospital, decreased morbidity, attenuated stress, lower in-hospital cost, and accelerated recovery in various types of surgery during the perioperative period.

ERAS is a pathway involving multidisciplinary efforts including surgery, anesthesia, psychology, nutrition, and nursing support. The outcomes of the ERAS pathway are affected by multiple aspects and anesthesia is just one of them. However, anesthesia does play an important role as it impacts the short-term recovery, patient satisfaction, in-hospital complications such as the incidences of postoperative cognitive dysfunction (POCD) and PONV [18, 19], and in some cases, even long-term outcomes [20, 21]. In this study, we develop an ERAS pathway with the emphasis on minimizing the residual effects of anesthetics by using short-acting agents such as remifentanil, propofol, or desflurane so as to achieve rapid recovery. This combination regimen has been reported to facilitate early recovery without increasing PONV and pain [22]. To prevent the fast onset of postoperative pain because of the cessation of intraoperative remifentanil infusion, generous wound infiltration with 0.2% ropivacaine will be given, in order to avoid the use of long-acting opioids and facilitate early recovery.

By conducting this study, we hope to introduce the concept of ERAS to surgeons, anesthesiologists, nurses, and other allied health professionals. The ERAS pathway requires contributions from all of them. For the comparison group, there is no specific protocol regarding the conventional standard of care in each center, because we do not need to set up a standard “conventional” process to adjust their clinical practice only to make a comparison group with uniform standards. In contrast, for the ERAS group, the pathway is set up strictly with considerations of various aspects. Therefore, the implementation of this study requires deep understanding of ERAS and good collaboration among surgeons, anesthesiologists, ward nurses, and other allied health professionals.

With the hypothesis that ERAS could reduce the LOS while not increasing complications and in-hospital cost when compared with the current clinical practice, we also focus on whether the ERAS pathway could improve the patient’s quality of life or save medical resources. The outcome is to reflect these areas of interest. The primary outcome is LOS in hospital, which is affected by multiple factors such as preoperative preparation, optimization of patient’s pre-morbidities, perioperative anesthetic management, and in-hospital postoperative complications etc. It is an important index relevant to both patient’s quality of life and medical cost. In addition, hospital LOS is not only one of the most concerned outcomes by physicians, but also an index that governments of different countries are attempting to reduce in order to decrease healthcare costs [23]. Secondary outcomes including 30-day mortality, incidence of in-hospital complications, NRS for postoperative pain, total in-hospital cost, and 30-day readmission rate are all related to our hypothesis. Moreover, we divided the in-hospital complications into five grades to elaborate the severity of complications with a clear statement.

In summary, this trial is designed to test the hypothesis that the ERAS pathway could be superior to conventional clinical practice in reducing LOS without increasing the incidence of complications or medical cost.

## Trial status

The protocol version number is V2.0 and the date of this version is 26 January 2019. The anticipated starting date of recruitment is 1 August 2019. Recruitment is expected to be completed in December 2021.

## Supplementary information

**Additional file 1.** SPIRIT 2013 Checklist: Recommended items to address in a clinical trial protocol and related documents.

## Data Availability

The individual participant data and related materials will be uploaded in the IPD sharing platform to enable data sharing and will be available after the researcher’s agreement on reasonable request.

## Abbreviations

ASA: American Society of Anesthesiologists
BIS: Bispectral Index
CRS: Central Randomization System
ERAS: Enhanced recovery after surgery
LOS: Length of stay
NRS: Numerical rating scales
PACU: Post-anesthesia care unit
POCD: Postoperative cognitive dysfunction
PONV: Postoperative nausea and vomiting
SPSS: Statistic Package for Social Science
THA: Total hip arthroplasty
TKA: Total knee arthroplasty

## References

[1] Kurtz S, Ong K, Lau E, Mowat F, Halpern M (2007). Projections of primary and revision hip and knee arthroplasty in the United States from 2005 to 2030. J Bone Joint Surg Am..

[2] Kehlet H (1997). Multimodal approach to control postoperative pathophysiology and rehabilitation. Br J Anaesth.

[3] Geltzeiler CB, Rotramel A, Wilson C, Deng L, Whiteford MH, Frankhouse J (2014). Prospective study of colorectal enhanced recovery after surgery in a community hospital. JAMA Surg.

[4] Li S, Zhou K, Che G, Yang M, Su J, Shen C, Yu P (2017). Enhanced recovery programs in lung cancer surgery: systematic review and meta-analysis of randomized controlled trials. Cancer Manag Res.

[5] Wainwright TW, Immins T, Middleton RG (2016). Enhanced recovery after surgery (ERAS) and its applicability for major spine surgery. Best Pract Res Clin Anaesthesiol.

[6] Soffin EM, YaDeau JT (2016). Enhanced recovery after surgery for primary hip and knee arthroplasty: a review of the evidence. Br J Anaesth.

[7] Hollman F, Wolterbeek N, Veen R (2015). Risk Factors for Postoperative Urinary Retention in Men Undergoing Total Hip Arthroplasty. Orthopedics.

[8] Berninger MT, Friederichs J, Leidinger W (2018). Effect of local infiltration analgesia, peripheral nerve blocks, general and spinal anesthesia on early functional recovery and pain control in total knee arthroplasty. BMC Musculoskelet Disord.

[9] Mandel JE (2014). Considerations for the use of short-acting opioids in general anesthesia. J Clin Anesth.

[10] Chow S, Shao J, Wang H. Sample Size Calculations in Clinical Research (2nd edn). Boca Raton: Chapman & Hall/CRC Biostatistics Series (Chapman and Hall/CRC); 2008. p. 61.

[11] Christelis N, Wallace S, Sage CE (2015). An enhanced recovery after surgery program for hip and knee arthroplasty. Med J Aust.

[12] Wijk L, Franzen K, Ljungqvist O, Nilsson K (2014). Implementing a structured Enhanced Recovery After Surgery (ERAS) protocol reduces length of stay after abdominal hysterectomy. Acta Obstet Gynecol Scand.

[13] Pędziwiatr M, Kisialeuski M, Wierdak M, Stanek M, Natkaniec M, Matłok M, Major P, Małczak P, Budzyński A (2015). Early implementation of Enhanced Recovery After Surgery (ERAS®) protocol - Compliance improves outcomes: A prospective cohort study. Int J Surg.

[14] Khan S, Gatt M, MacFie J (2009). Enhanced recovery programmes and colorectal surgery: does the laparoscope confer additional advantages?. Color Dis.

[15] Ljungqvist O, Scott M, Fearon KC (2017). Enhanced Recovery After Surgery: A Review. JAMA Surg.

[16] Coolsen MM, van Dam RM, van der Wilt AA, Slim K, Lassen K, Dejong CH (2013). Systematic review and meta-analysis of enhanced recovery after pancreatic surgery with particular emphasis on pancreaticoduodenectomies. World J Surg.

[17] Liu F, Wang W, Wang C, Peng X (2018). Enhanced recovery after surgery (ERAS) programs for esophagectomy protocol for a systematic review and meta-analysis. Medicine (Baltimore).

[18] Tong D, Chung F, Wong D (1997). Predictive factors in global and anesthesia satisfaction in ambulatory surgical patients. Anesthesiology.

[19] Homburger JA, Meiler SE (2006). Anesthesia drugs, immunity, and long-term outcome. Curr Opin Anaesthesiol.

[20] Dürsteler C, Mases A, Puig MM (2008). Universal PONV prophylaxis in general anesthesia: should we consider its immediate implementation?. Anesth Analg.

[21] Newman S, Stygall J, Hirani S, Shaefi S, Maze M (2007). Postoperative cognitive dysfunction after noncardiac surgery: a systematic review. Anesthesiology.

[22] Song D, White PF (1999). Remifentanil as an adjuvant during desflurane anesthesia facilitates early recovery after ambulatory surgery. J Clin Anesth.

[23] Shayne M, Culakova E, Poniewierski MS, Dale DC, Crawford J, Wogu AF, Lyman GH (2013). Risk factors for in-hospital mortality and prolonged length of stay in older patients with solid tumor malignancies. J Geriatr Oncol.

